# Contributing Factors to the Burden on Primary Family Caregivers of Stroke Survivors in South Korea

**DOI:** 10.3390/ijerph20032760

**Published:** 2023-02-03

**Authors:** Bo Mi Kwon, Hyun Haeng Lee, Min Kyun Sohn, Deog Young Kim, Yong-Il Shin, Gyung-Jae Oh, Yang-Soo Lee, Min Cheol Joo, So Young Lee, Min-Keun Song, Junhee Han, Jeonghoon Ahn, Won Hyuk Chang, Jongmin Lee, Yun-Hee Kim

**Affiliations:** 1 Department of Rehabilitation Medicine, Konkuk University School of Medicine, Seoul 05030, Republic of Korea; 2 Department of Rehabilitation Medicine, College of Medicine, Chungnam National University, Daejeon 35015, Republic of Korea; 3 Department and Research Institute of Rehabilitation Medicine, Yonsei University College of Medicine, Seoul 03722, Republic of Korea; 4 Department of Rehabilitation Medicine, School of Medicine, Pusan National University Yangsan Hospital, Pusan National University, Yangsan-si 50612, Republic of Korea; 5 Department of Preventive Medicine, School of Medicine, Wonkwang University, Iksan 54538, Republic of Korea; 6 Department of Rehabilitation Medicine, Kyungpook National University School of Medicine, Kyungpook National University Hospital, Daegu 41566, Republic of Korea; 7 Department of Rehabilitation Medicine, School of Medicine, Wonkwang University, Iksan 54538, Republic of Korea; 8 Department of Rehabilitation Medicine, Jeju National University Hospital, Jeju National University College of Medicine l, Jeju-si 63241, Republic of Korea; 9 Department of Physical and Rehabilitation Medicine, Chonnam National University Medical School, Gwangju 61469, Republic of Korea; 10 Department of Statistics, Hallym University, Chuncheon-si 24252, Republic of Korea; 11 Department of Health Convergence, Ewha Womans University, Seoul 03760, Republic of Korea; 12 Department of Physical and Rehabilitation Medicine, Center for Prevention and Rehabilitation, Samsung Medical Center, Heart Vascular Stroke Institute, Sungkyunkwan University School of Medicine, Suwon 06351, Republic of Korea; 13 Department of Health Science and Technology, Department of Medical Device Management and Research, Department of Digital Healthcare, SAIHST, Sungkyunkwan University, Suwon 06351, Republic of Korea

**Keywords:** primary caregiver, caregiver burden, neurological impairment, functional dependence, depression, intimacy

## Abstract

The purpose of this study is to identify the factors associated with the burden on primary family caregivers of stroke patients at home without care services. For this study, the Korean Stroke Cohort for Functioning and Rehabilitation (KOSCO) data were used. Of the total 8010 caregivers, 1133 family caregiver burden was assessed with the shortened Caregiver Burden Inventory (CBI) 3 months after stroke. Patient and caregiver-related factors affecting the heavier burden of caregivers were identified by comparing the heavier caregiver burden group and the lighter caregiver burden group, which divided according to the CBI scores. The 719 (63.5%) family caregiver cared for patients at home without care services. Logistic regression analysis showed that four or more comorbidities (*p* = 0.002), neurological impairment at early onset (*p* < 0.001), dependence on daily life (*p* < 0.001), aphasia (*p* = 0.024), and depression(*p* < 0.001) were associated with a heavier burden of care. According to the shortened CBI, caregivers tended to be concerned more about psychological stress than physical strain. The findings suggest the importance of proactively guiding the emotional support services to caregivers who are at high risk of the heavier burden of patient care.

## 1. Introduction

Stroke is a major cause of long-term disability [1,2]. In Korea, the incidence of stroke is gradually increasing with the aging of the population [3]. Many stroke survivors return to the community with functional impairments after receiving acute treatment and rehabilitation [3,4]. Even after discharge, informal caregivers provide daily living support to stroke survivors according to their level of functional independence [5,6]. Many informal caregivers experience negative health effects such as depression, anxiety, and general illness while helping stroke survivors with dysfunction [6,7]. Previous studies have shown that from 25 to 54% of informal caregivers experience significant burdens. The caregiver burden is relatively high in the first 3 months after stroke [6].

Various patient-related and caregiver-related factors that affect caregiver burdens have been explored [6]. The patient-related factors include patients’ daily dependence, anxiety, depression and caregiver-related factors were presented as female, depression, anxiety, lack of a sense of coherence, daughters-in-law, and the time and effort spent in caring responsibilities [6,8]. Furthermore, the socioeconomic and care cultural factors are also significant factors influencing the high caregiver burden [6,9,10,11]. In previous studies, the lack of tangible care support was shown to affect the psychological burden on caregivers [12,13]. In addition, unemployment status after stroke was found to be a factor that increases the burden of care [10]. On the other hand, in the socio-cultural aspect, the patriarchal care culture has affected the burden of women and daughters-in-law as primary caregivers [10,11,14]. However, the significance of factors may be altered by changes in public health services and sociodemographic backgrounds. We need to identify the factors affecting the burden of stroke care after the introduction of long-term care services in Korea.

In Korea, long-term care services have been implemented nationwide since 2008 to ease caregivers’ burden. Long-term care services have been confirmed to be effective in alleviating the burden of care for the economically vulnerable and in reducing family support obligations [15]. Long-term care services include in-facility and home-based services, such as visiting care, bathing, nursing, and day-night care. These were provided according to the patient’s functional dependence. However, since patients with subacute stroke are in the process of recovery, it is difficult to evaluate their continued functional dependence, which limits the provision of long-term care services. This is expected to impact the burden on family caregivers caring for stroke survivors at home.

Therefore, this study aimed to identify the factors associated with the burden of caregivers who are living with patients without long-term care services, based on nationwide stroke cohort data. In addition, through the care burden questionnaire, we will verify the burdensome items about which caregivers who experience heavier burdens frequently complain. It will be an important basis for the effective and concerted delivery of limited resources for health care services to caregivers with heavy burdens.

## 2. Materials and Methods

### 2.1. Data and Sampling

This study is based on the nationwide cohort data. The Korean Stroke Cohort for Functioning and Rehabilitation (KOSCO) study includes the data from patients admitted to university hospitals located in nine regions of South Korea for the care of first-ever acute stroke. The study is designed to investigate the factors influencing functional impairment, neurological recovery, and long-term quality of life of stroke survivors within 10 years after stroke. For the adequate level of inter-rater reliability, all raters underwent a standardized training program every 3 months throughout the course of the study [16].

We included the data from stroke patients and their primary family caregivers. The inclusion criteria for the patients were: (1) 19 years of age or older, (2) first-ever stroke patient, and (3) had primary caregivers who offered 3 months of caregiving without payment. The exclusion criteria were: (1) patients with a prior history of stroke, (2) patients with transient ischemic attack, or traumatic intracranial hemorrhage, and (3) foreigners.

All patients or their legally authorized representatives provided written informed consent before being included in this study. The study protocol was approved by the institutional review board of each participating hospital (KUH1180017).

### 2.2. Grouping by the Level of Caregiver Burden

Caregiver burden was measured 3 months after stroke (i.e., 3 months of caregiving) using the shortened Caregiver Burden Inventory (CBI) that was adapted for the KOSCO Caregiver burden survey. The CBI is one of the most widely used validated tools for quantifying the burden of informal caregivers [17]. We constructed the shortened CBI by selecting the most representative and pertinent 13 items from the original CBI and adding two economic-status-related questions. Each item of the shortened CBI was rated on a 5-point scale ranging from 0 (never) to 4 (nearly always), and the total scale score ranged from 0 to 60. We divided the caregivers into two groups; the top 50% (heavier caregiver burden) and the bottom 50% (lighter caregiver burden), based on the total shortened CBI scores.

#### 2.2.1. Patient-Related Factors and the Burden of Care

Patient characteristics such as age, sex, weighted index of comorbidity (WIC), and neurological impairment were assessed within 7 days from the onset of stroke. The patients’ post-stroke functional impairment was assessed 3 months after the onset of stroke. Specifically, we assessed the patient-related factors that can potentially influence the caregiver burden using the National Institute of Health Stroke Scale (NIHSS) for neurological impairment [18], Fugl-Meyer Assessment (FMA) for motor dysfunction [19], modified Rankin Scale (mRS) for functional dependence [20], American Speech-Language-Hearing Association National Outcomes Measurement System Swallowing Scale (ASHA-NOMS) for swallowing dysfunction [21], the Korean version of the Frenchay Aphasia Screening Test (K-FAST) for language disability [22], and Korean Mini-Mental State Examination (K-MMSE) for cognitive impairment [23].

The functional level of the patients was judged based on the following criteria: the mRS value of 0–1 range indicates independence in daily living, 2 indicates dependency only in the instrumental activity of daily living (IADL), and 3–5 range indicates dependency in both basic activity of daily living (BADL) and IADL. Cognitive impairment was determined based on the MMSE score of ≤23 [23]. Tube feeding or oral feeding with dietary adjustments was considered a dietary restriction. Aphasia was determined based on the K-FAST of <20 [22]. Depression was determined based on the Geriatric Depression Scale (GDS) value of ≥10 [24].

#### 2.2.2. Caregiver-Related Factors and the Burden of Care

The caregiver-related factors such as age, sex, relationship with the patient, level of intimacy with the patient, receiving long-term care services, education level, types of health insurance, and availability of ancillary caregivers were assessed after 3 months of caregiving. Primary family caregivers include spouses, children, daughters-in-law, grandchildren, and cousins. The level of intimacy with the patient was split into high and low (to moderate) categories. Examples of the long-term care services included visiting nursing services, daycare centers, long-term care facilities, and rehabilitation facilities. A high school diploma or higher was considered as a high level of education. Types of health insurance were health insurance payer and benefit recipient depending on the economic status. Receiving long-term care services, and availability of ancillary caregivers were assessed based on “yes” or “no” responses.

### 2.3. Statistical Analysis

We used the *t*-test, Mann-Whitney U test, and Chi-squared test to examine the difference in each study variable between the heavier and lighter caregiver burden groups. A logistic regression analysis was conducted to examine the factors associated with the incidence of high caregiver burden. To construct a model for the logistic regression analysis, we introduced the study variables showing significant differences with a *p*-value < 0.01 between the heavier and lighter caregiver burden groups as independent variables. The level of statistical significance was set at *p* < 0.05. We used the R programming language (ver. 3.4.4; https://www.r-project.org/, accessed on 24 June 2021) for statistical analysis and visualization.

## 3. Results

### 3.1. Characteristics of Patients and Caregivers

**Figure 1:**
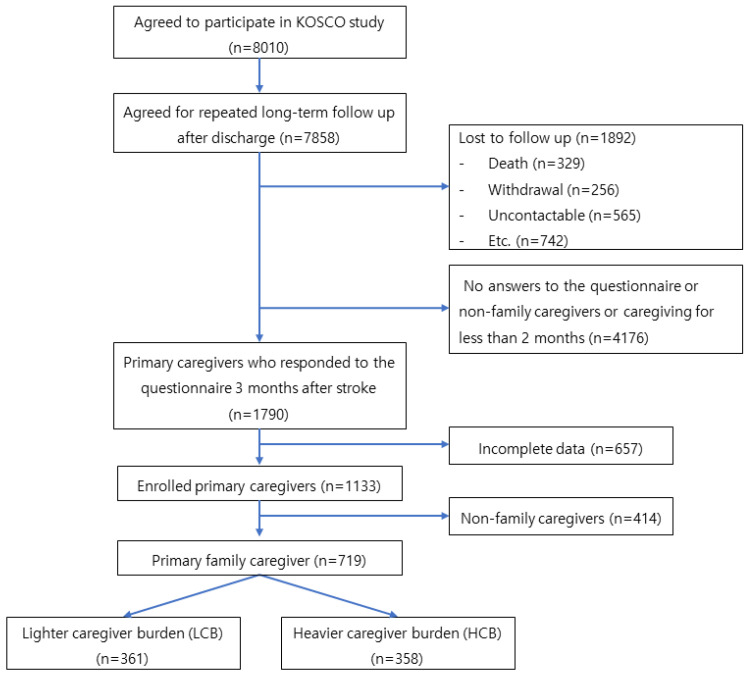
Flow chart of caregivers who participated in the survey. The survey to assess the caregiver burden was administered three months after stroke. The primary caregivers who responded to the questionnaire had continued to care for the patients for three months after stroke. The caregivers were divided into two groups based on the shortened Caregiver Burden Inventory (CBI) scores. Those in the top 50% were classified as heavier caregiver burden group and those in the bottom 50% were classified as lighter caregiver burden group. KOSCO = Korean Stroke Cohort for Functioning and Rehabilitation.

Of the total 8010 patients, 7858 consented to long-term follow-up (Figure 1). We excluded patients if they missed follow-up or provided incomplete data, as well as if their caregivers did not respond to questionnaires or cared for the patients for less than 3 months. Among the total primary caregivers, 1133 of them cared for the patients for 3 months after the onset of stroke. The numbers of caregivers in the Heavier Caregiver Burden Group (HBG; top 50%) and the Lighter Caregiver Burden Group (LBG; bottom 50%) were respectively 556 and 577.

The mean age of the patients (*n* = 1133) was 67.1 ± 11.6 years, and 675 (59.6%) of them were male. (Table 1) The initial NIHSS score mean was 4.4 ± 4.6, which was mild to moderate neurological impairment. The number of patients who were dependent on their caregivers for BADL 3 months after stroke was 262 (23.1%).

The mean age of the primary caregivers (*n* = 1133) was 54.9 ± 13.7 years, and 742 (65.5%) of them were female (Table 1). Primary family caregivers 898 (79.3%) cared for the patients at home without long-term care services. The mean CBI score for 3 months of caregiving was 15.5 ± 13.5. Some 235 (20.7%) caregivers used long-term care services such as home visits, daycare centers, and long-term care facilities.

### 3.2. Patient- and Caregiver-Related Factors Associated with the Heavier Burden of Family Caregivers Who Care for the Patients at Home without Care Services

Of the total 1133 caregivers, 898 were family caregivers who cared for the patients at home without care services (Table 2). The following factors were significantly associated with the heavier burden for them: High initial NIHSS score (*p* < 0.001), WIC equal or greater than 5 (*p* = 0.002), mRS score equal or greater than 3 (*p* < 0.001), aphasia (*p* = 0.024), and depression (*p* < 0.001). The probability of being in the heavier caregiver burden group was 3.217 times greater when the patients’ dependency in BADL was higher (95% CI = 1.680–6.395, *p* < 0.001) and 4.027 times greater when there were four or more comorbidities (95% CI = 1.731–10.529, *p* < 0.001). However, the patients’ FMA upper extremity motor dysfunction and cognitive impairment was not independently and significantly associated with a heavier burden of care. On the other hand, the patients’ aphasia (OR = 1.562, 95% CI = 1.059–2.305, *p* = 0.024) and depression (OR = 2.543, 95% CI = 1.616–4.059, *p* < 0.001) was significantly associated with the increased probability of being in the heavier caregiver burden group.

Caregiver-related factors like low intimacy with the patient (*p* = 0.022) and being a benefit recipient of health insurance (*p* = 0.035) were found to be significantly associated with the heavier burden of care. However, they were not independently and significantly associated with the heavier caregiver burden after controlling for other variables.

### 3.3. Difference in Responses Rate for the Shortened CBI Items between the Heavier and Lighter Caregiver Burden Groups

**Figure 2:**
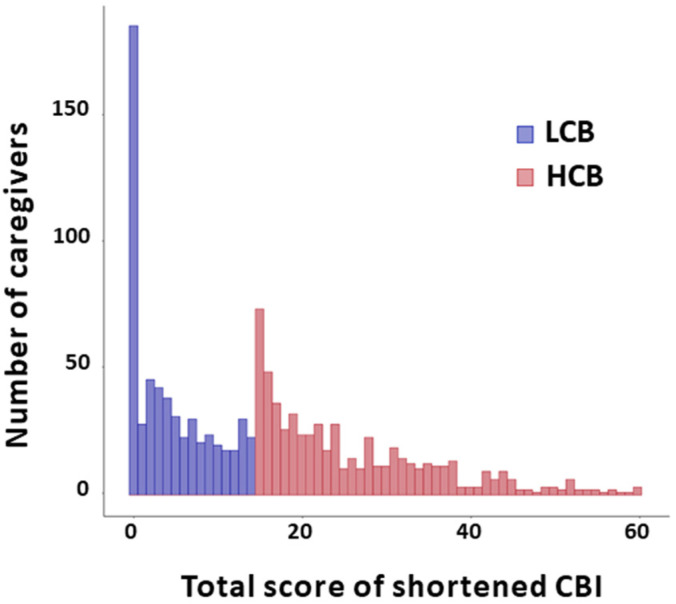
Responses to the shortened CBI items in the primary caregivers who cared for the stroke patients at home without care services. The total score distribution of the shortened CBI for all primary caregivers. Low caregiver burden group’s CBI score range was 0–15 and the heavier caregiver burden group’s CBI score range was 16–60.

**Figure 3:**
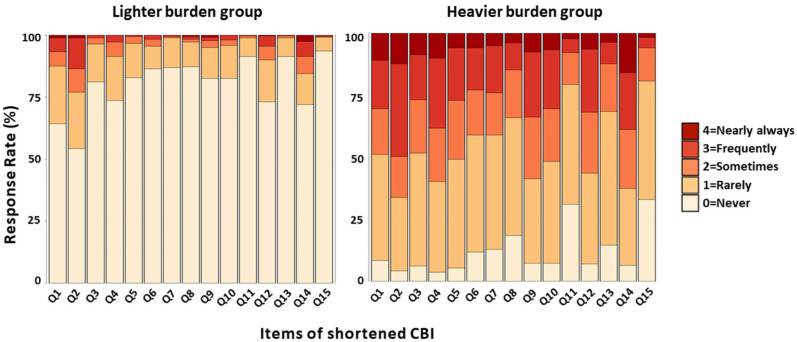
A difference in response rate for shortened CBI items in the primary caregivers who cared for the stroke patients at home without care services. There was a notable difference in response rate for each shortened CBI item.

The average caregiver burden for primary family caregivers who cared for the patients for 3 months after stroke was 14.8 ± 12.8. The mean score of the shortened CBI scores 4.47 ± 4.61 for the lighter caregiver burden group and 25.16 ± 9.64 for the heavier caregiver burden group. (Figure 2) A difference in response rate for each shortened CBI item was found. There was a significant difference in response rates in all sub-items between the two groups. (Figure 3) The top three items with the largest difference in response distribution between the two groups were: ‘I have to watch my care receiver constantly’(Q2), ‘My social life has suffered’(Q4), and ‘My caregiving efforts are not appreciated by others in my family’(Q9).

## 4. Discussion

We identified the factors associated with the heavier burden on caregivers caring for patients at home without care services in the first three months after the stroke in a large population. We found that 898 (79.3%) of the 1133 family caregivers took care of patients without care services. Patient-related factors affecting the heavier caregiver burden were the high initial NIHSS score (*p* < 0.001), WIC greater than 5 (*p* = 0.002), mRS score of 3 or greater (*p* < 0.001), aphasia (*p* = 0.028), and depression (*p* < 0.001). None of the caregiver-related factors independently affected the caregiver burden. Meanwhile, in the caregiver burden questionnaire, the items with the largest standardized mean difference between the two groups were: ‘I have to watch my care receiver constantly’(Q2), ‘My social life has suffered’(Q4), ‘My caregiving efforts are not appreciated by others in my family’(Q9), and ‘I feel angry about my interactions with the patient’ (Q14).

Regarding the population characteristics, the age difference between the patient and caregiver was approximately 12 years or more. This age gap is larger than the ones reported in previous studies on family caregivers caring for stroke patients [10,25,26,27]. This might be attributed to the broad range of family caregivers including children, daughters-in-law, and grandchildren. As a result of identifying family members who were mainly in charge of caring, most were spousal caregivers (56.1%), followed by sons and daughters (34%), daughters-in-law (4.5%), and others (5.4%). Contrary to the results of previous studies on the burden of stroke care in Korea, [10] female and daughters-in-law as primary caregivers were not related to the burden on caregivers. Although family-centered care is the mainstream in Korea, [28] it suggests that the responsibility for care, which was concentrated on women, has been eased according to socio-cultural changes.

Caring for patients with multiple comorbidities (i.e., WIC 5 or greater) at home without care service increased the odds ratio by 3.262 to be included in a heavier caregiver burden. Stroke patients with comorbidities require long-term treatment and symptom management. Caregivers were able to receive assistance from medical experts in managing patients’ comorbidities in medical institutions. However, after discharge, it becomes the caregiver’s responsibility to control and manage the patient’s comorbidities. Regardless of caregiver efforts, many comorbidities can lead to reduced function in patients. In addition, comorbidities such as hypertension, diabetes, and congestive heart failure are major risk factors that increase the recurrence of stroke, [29] which will put a great psychological burden on caregivers. Therefore, it is necessary to relieve the caregiver’s burden through post-discharge-related services for stroke patients who need continuous control and management of comorbidities.

We confirmed that most stroke patients included in this study had mild to moderate neurological impairments. Nevertheless, the functional dependence of patients caused by neurological disorders places a heavy burden on caregivers without care services. Adams et al. reported that a 1-point increase in the NIHSS score in the acute phase was associated with a 17% decrease in functional independence after 3 months [30]. The patient dependence is known to interfere with caregivers’ social interaction and increase the psychological burden [27]. This was reflected in the difference in the high response rate in the care burden survey (i.e., Q2. I have to watch my care receiver constantly, Q4. My social life has suffered). This suggests the importance of early psychological counseling support for caregivers who care for highly dependent patients at home so that they do not fall into anxiety or depression.

Aphasia and depression are independent factors that contribute to a heavier burden on the caregiver. These cause communication difficulties between patients and caregivers and reduce the quality of their relationships [31,32]. The difficulty of interacting with patients puts a considerable psychological burden on the person caring. This is especially burdensome for caregivers who lack communication skills and coping strategies [27,33]. This is also shown in the relatively high negative response rate to ‘I feel angry about my interactions with the patient’ (Q14) of the care burden questionnaire. Previous studies have suggested various coping strategies to address this caregiver burden [34,35,36]. A meta-analysis reported that dyadic psychoeducational interventions are most effective in improving the caregiver burden. In particular, it is effective for interventions to begin in hospitals in preparation for discharge [37]. Therefore, considering that caring for patients with aphasia and depression at home without care services contributes to the heavy burden of family care, providing psychoeducational interventions along with discharge education is expected to be effective in reducing the burden of care for caregivers of patients with subacute stroke.

This study has some limitations to be noted. First, focus was placed on the patients with relatively mild or moderate neurological impairment after stroke. Patients with severe neurological problems were likely to be transferred to a rehabilitation hospital or long-term care facility after acute stroke treatment. Moreover, most of them tended to be cared for by paid long-term caregivers, and ended up being excluded from this study. As a result, only a small number of patients with severe dysfunction were included in the present study and this may lead to the underestimation of the caregiver burden. Second, we were not able to examine the effects of caregivers’ depression and anxiety even though they were known as major contributing factors to high caregiver burden in a previous study [6]. Studies need to examine the role of psychological wellbeing and mental health of caregivers on their perceived and experienced burden of caregiving. Third, we could not confirm the inter- and intra-rater reliability of the shortened CBI to measure caregiver burden. Nevertheless, we secured reliability by conducting regular training sessions so that the evaluators could reach some degree of consensus regarding the level of measurement.

## 5. Conclusions

Multiple factors are systematically associated with the burden of family caregiver caring for patients at home without care services. Many comorbidities and a high dependence of the stroke patients caused by neurological impairment were associated with the caregivers’ heavier burden of caring. Moreover, communication problems with the patient may cause emotional stress exacerbating the burden of care. According to the findings from the CBI, caregivers’ complaints in caregiving were concerned more about psychological stress than physical strain. Our results suggest the need for psychological interventions to mitigate the heavier burden of caregivers of subacute stroke. It should be provided along with the patient care education programs for those caregivers who are at heightened risk of a heavier burden of patient care.

## Figures and Tables

**Table 1:** Characteristics of the enrolled patients and primary caregivers.

Variables	Total (*n* = 1133)
Patients	
Age (years) [range]	67.1 ± 11.6 [20–95]
Sex [*n*]	
Male	675 (59.6%)
Female	458 (40.4%)
WIC [*n*]	
1–4	1080 (95.3%)
≥5	53 (4.7%)
Initial NIHSS (score) [range]	4.4 ± 4.6 [0–34]
mRS [*n*]	
0–1	672 (59.3%)
2	199 (17.6%)
3–5	262 (23.1%)
FMA (affected side) [range]	89.1 ± 21.6 [0–100]
Cognitive impairment [*n*]	
Yes	337 (29.7%)
no	796 (70.3%)
Aphasia [*n*]	
yes	373 (32.9%)
no	760 (67.1%)
Types of feeding [*n*]	
No restriction	937 (82.7%)
Tube feeding or oral feeding with restriction	196 (17.3%)
Depression [*n*]	
yes	253 (22.3%)
no	880 (77.7%)
Caregivers	
Age (years) [range]	54.9 ± 13.7 [19–92]
Sex [*n*]	
Male	391 (34.5%)
Female	742 (65.5%)
CBI total score [range]	15.5 ± 13.5 [0–60]
Living with the patient [*n*]	
Yes	821 (72.5%)
No	312 (27.5%)
Level of intimacy [*n*]	
good	927 (81.8%)
medium or bad	206 (18.2%)
Receiving long-term care services [*n*]	
Yes	235 (20.7%)
No	898 (79.3%)
Level of education [*n*]	
Low	374 (33.0%)
High	759 (67.0%)
Types of health insurance [*n*]	
health insurance payer	1049 (92.6%)
benefit recipient	35 (3.1%)
other	49 (4.3%)
Availaibility of ancillary caregiver [*n*]	
Yes	402 (35.5%)
no	731 (64.5%)

Abbreviations: M, male; F, female; BMI, body mass index; WIC, Weighted Index of Comorbidity; NIHSS, National Institutes of Health Stroke Scale; FMA, Fugl-Meyer Assessment; mRS, modified Rankin Scale.

**Table 2:** Identification of patient- and caregiver-related factors associated with the heavier burden of primary family caregivers who care for the patients at home without care services.

Variables	Total (*n* = 719)	Lighter Caregiver Burden Group (*n* =361)	Heavier Caregiver Burden Group (*n* = 358)	Unadjusted OR		Adjusted OR	
				OR (95% CI)	*p*-Value	OR (95% CI)	*p*-Value
Patients							
Age (years)	65.6 ± 11.5	65.2 ± 11.8	66.1 ± 11.2	1.007	0.315	NA	NA
	[20–95]	[20–94]	[26–95]	(0.994–1.020)			
Sex [*n*]							
Male	469 (65.2%)	248 (68.7%)	221 (61.7%)	1.000			
Female	250 (34.8%)	113 (31.3%)	137 (38.3%)	1.361	0.050	NA	NA
				(1.000–1.853)			
WIC [*n*]							
1–4	685 (95.3%)	354 (98.1%)	331 (92.5%)	1.000			
≥5	34 (4.7%)	7 (1.9 %)	27 (7.5 %)	4.125	0.001	4.027	0.002
				(1.870–10.401)		(1.731–0.529)	
Initial NIHSS (score) [range]	3.9 ± 4.1 [0–27]	3.0 ± 3.2	4.8 ± 4.6	1.128	<0.001	1.079	<0.001
		[0–27]	(1.082–1.180)		(1.034–1.129)		
mRS [*n*]							
0–1	492 (68.4%)	429 (75.3%)	260 (43.8%)	1.000		NA	
2	123 (17.1%)	86 (15.1%)	116 (19.6%)	2.463	<0.001	3.217	NA < 0.001
3–5	104 (14.5%)	55 (9.6%)	217 (36.6%)	(1.647–3.716)	<0.001	(1.680–6.395)	
				7.795			
				(4.606–13.832)			
FMA (affected side) (score) [range]	94.3 ± 13.5	97.4 ± 7.4	91.3 ± 17.1	0.955	<0.001	NA	NA
	[8–100]	[39–100]	[8–100]	(0.937–0.970)			
Cognitive impairment (yes) [*n*]	177 (24.6%)	58 (16.1 %)	119 (33.2 %)	2.601	<0.001	NA	NA
				(1.828–3.735)			
Aphasia (yes) [*n*]	193 (26.8%)	67 (18.6%)	126 (35.2%)	2.383	<0.001	1.562	0.024
				(1.697–3.370)		(1.059–2.305)	
Types of feeding [*n*]				1.000			
No restriction	613 (85.3%)	313 (86.7%)	300 (83.8%)	1.261			
Tube feeding or oral Feeding with restriction	106 (14.7%)	48 (13.3%)	58 (16.2%)	(0.834–1.913)	0.273	NA	NA
Depression (yes) [*n*]	142 (19.7%)	34 (9.4%)	108 (30.2%)	4.155	<0.001	2.543	<0.001
				(2.760–6.395)		(1.616–4.059)	
Caregivers							
CBI total score	14.8 ± 12.8	4.47 ± 4.61	25.16 ± 9.64				
	[0–59]	[0–14]	[15–59]				
Age (years)	58.5 ± 13.6	58.0 ± 14.2 [19–92]	58.9 ± 13.0	1.005	0.347	NA	NA
	[19–92]		[23–85]	(0.994–1.016)			
Sex [*n*]				1.000			
Male	245 (34.1%)	126 (34.9%)	119 (33.2%)	1.077			
Female	474 (65.9%)	235 (65.1%)	239 (66.8%)	(0.791–1.467)	0.638	NA	NA
Level of intimacy [*n*]							
Good	575 (80.0%)	301 (83.4%)	274 (76.5%)	1.000			
Medium or bad	144 (20.0%)	60 (16.6%)	84 (23.5%)	1.538 (1.065–2.232)	0.022	NA	
Level of education [*n*]							
Low	292 (40.6%)	145 (40.2%)	147 (41.1%)	1.000			
High	427 (59.4%)	216 (59.8%)	211 (58.9%)	0.964	0.807	NA	NA
				(0.715–1.298)			
Types of health insurance [*n*]		338 (93.6%)					
Health insurance payer	662 (92.1%)		324 (90.5%)	1.000			
Benefit recipient	22 (3.1%)	6 (1.7%)		2.782	0.035	NA	NA
Other	35 (4.9%)	17 (4.7%)	16 (4.5%)	(1.129–7.833)	0.774	NA	NA
			18 (5.0%)	1.105(0.557–2.197)			
Availability of ancillary caregiver (yes) [n]	>192 (26.7%)	>91(25.2%)	>101 (28.2%)	>1.166 (0.838–1.625)	>0.363	>NA	>NA

Abbreviations: OR, odd ratios; BMI, body mass index; WIC, Weighted Index of Comorbidity; NIHSS, National Institutes of Health Stroke Scale; FMA, Fugl-Meyer Assessment; mRS, modified Rankin Scale; NA, not applicable. Odds ratio obtained through Haldane-Anscombe correction.

## Data Availability

The datasets generated during and/or analysed during the current study are available from the corresponding author on reasonable request.
